# Ultralight, Mechanically Enhanced, and Thermally Improved Graphene-Cellulose-Polyethyleneimine Aerogels for the Adsorption of Anionic and Cationic Dyes

**DOI:** 10.3390/nano12101727

**Published:** 2022-05-18

**Authors:** Xiuya Wang, Pengbo Xie, Lan He, Yuwei Liang, Liang Zhang, Yuanyuan Miao, Zhenbo Liu

**Affiliations:** Key Laboratory of Bio-Based Material Science and Technology of Ministry of Education, Northeast Forestry University, Harbin 150040, China; wangxiuya2019@nefu.edu.cn (X.W.); xiepengbo@nefu.edu.cn (P.X.); namehelan@nefu.edu.cn (L.H.); lyw0824@nefu.edu.cn (Y.L.); zhliang17@nefu.edu.cn (L.Z.)

**Keywords:** graphene, cellulose, polyethyleneimine, aerogel, adsorbent

## Abstract

Graphene-cellulose-polyethyleneimine aerogels (GA-MCC-PEI) were prepared using a simple, environmentally friendly method to remove anionic and cationic dyes in water. Graphene-cellulose hydrogels were prepared using a hydrothermal method and then immersed in a polyethyleneimine aqueous solution for 48 h to obtain graphene-cellulose-polyethyleneimine hydrogels, which were then freeze-dried. The light and porous composite aerogels had a good compression resistance, and the maximum allowable pressure of the graphene-cellulose-polyethyleneimine aerogel with a cellulose content of 43% was 21.76 kPa, which was 827 times its weight. Adsorption of the anionic dye amaranth and the cationic dye methylene blue by the graphene-cellulose-polyethyleneimine aerogel was satisfactorily modeled using the Langmuir isothermal equation, indicating monolayer adsorption. When the cellulose content was 39%, the equilibrium adsorption capacities of the composite aerogel for amaranth and methylene blue were 369.37 mg/g and 237.33 mg/g, respectively. This graphene-cellulose-polyethyleneimine aerogel can be used to remove dye pollutants in water to maintain ecological balance, thus broadening the application space of aerogel materials, that is, as adsorbents in different environments.

## 1. Introduction

Dyes in water can adversely affect ecosystems and human health. Adsorption has become one of the most effective wastewater treatment methods because of its low cost, simple operation, and high efficiency [1,2,3]. Graphene aerogels are porous (porosity can reach more than 99%), which can provide sufficient transport channels for the diffusion of pollutants when they are adsorbed to improve the adsorption efficiency of adsorbents [4,5]. Graphene aerogels also have the advantages of strong flexibility, ultra-low density, porous structure, ideal specific surface area, and high adsorption capacity, and are able to be used as adsorbents in different environments [6,7]. However, owing to the van der Waals and π–π interactions between graphene sheets, irreversible stacking and agglomeration occur, which negatively affects the mechanical properties and pore structure of graphene aerogels, thus limiting their practical applications. Moreover, aerogels composed of a single material do not have multiple functions [8,9,10,11,12]. Therefore, other functional materials have been incorporated into graphene aerogels to address the above-mentioned problems. Multi-component composite aerogels with high mechanical strengths and adsorption capacities have high practical value.

Cellulose has numerous advantages, including good hydrophilicity, non-toxicity, biodegradability, biocompatibility, and low density. Importantly, cellulose has multiple binding sites on its surface for hydroxyl and hydrogen bonds, which can serve as linkage points with other polymers to form strong composite materials [13,14,15]. In graphene-cellulose composite aerogels, cellulose not only forms hydrogen bonds with graphene to enhance the mechanical properties of the composite but also acts as a spacer between graphene sheets to prevent the stacking of sheets stabilized by van der Waals and π–π interactions [13,14,15,16,17,18]. Zhang et al. used a bidirectional freeze-drying method to prepare cellulose-graphene aerogels with high elasticity and anisotropy and, furthermore, silanized the composite aerogels using chemical vapor deposition (CVD) [19]. A superhydrophobic cellulose-graphene aerogel (MCGA) was obtained. MCGA has a bidirectionally arranged porous structure and an ultra-light bulk density (5.9 mg/cm^3^). The modified aerogel can absorb oil from water, with an adsorption capacity as high as 80 to 197 times its own mass, surpassing most carbon-based aerogels.

Graphene-cellulose aerogels can be used to adsorb pollutants, such as dyes in water, via their dense porous structure. A simple, effective, and green method can be developed to prepare graphene-cellulose aerogels by which the efficiency of physical adsorption is improved and the adsorption capacity for anionic and cationic dyes is increased. The sensitivity of graphene-cellulose aerogel is a problem that needs to be solved for practical adsorption [20]. For this reason, we attempted to increase the adsorption capacity of composite materials by increasing the number and type of active sites. Graphene-cellulose aerogels were functionalized with amine groups, and cationic polyethyleneimine (PEI), which has high adhesion and adsorption capacities, was introduced into the composite aerogels [21,22,23]. PEI is a water-soluble polymer containing reactive groups (primary, secondary, and tertiary amines) that easily react with carboxyl and other functional groups. Moreover, it can electrostatically adsorb negatively charged pollutants and also form stable hydrogen bonds with cellulose and graphene, thus improving the adsorption and mechanical properties of composite aerogels [24,25,26]. Fang et al. prepared polyethyleneimine-cellulose nanofiber aerogels for the adsorption of Congo red dyes [27].

In this study, we modified graphene-cellulose aerogels with PEI and proposed a preparation method for graphene-cellulose-PEI aerogels. Cellulose prevents the stacking of graphene sheets, and PEI plays a role in optimizing the pore structure and adsorption capacity of the composite aerogel. Hydrogen bonding that is formed between the three materials is able to enhance the mechanical properties of the composites. This graphene-cellulose-PEI aerogel can be used to remove dye pollutants in water to maintain ecological balance, thus broadening the application space of aerogel materials, that is, as adsorbents in different environments.

## 2. Materials and Methods

### 2.1. Materials and Chemicals

Graphite crystals (99 wt.%) were obtained from Jiangsu Changjiade High-tech Carbon Materials Co., Ltd. (Changde, China). Hydrogen peroxide (H_2_O_2_, 30 wt.%) was provided by Tianjin Fuyu Fine Chemical Co., Ltd. (Tianjin, China). Sulfuric acid (H_2_SO_4_, 98 wt.%), potassium permanganate powder (KMnO_4_), and sodium nitrate (NaNO_3_) were purchased from Tianjin Comiou Chemical Reagent Co., Ltd. (Tianjin, China). Microcrystalline cellulose powder (MCC, particle size: 50 μm), polyethyleneimine (PEI), methylene blue (MB), amaranth (AM), sodium hydroxide (NaOH), and hydrogen chloride (HCl) were provided by Shanghai Aladdin Biochemical Technology Co., Ltd. (Shanghai, China). All chemicals were used as received without any further purification.

### 2.2. Preparation of Graphene Oxide (GO)

Preparation of graphene oxide (GO) by improved Hummers method [28], was as follows:

First of all, 46 mL concentrated sulfuric acid was kept at 4 °C by ice bath. Slowly add 2 g graphite powder, 1 g sodium nitrate, and 6 g potassium permanganate into concentrated sulfuric acid solution and stir evenly. Keep the mixed solution at 5–10 °C for 90 min. Then, the mixed solution was transferred to a water bath at 35–40 °C and taken out after 30 min. Add 92 mL deionized water to the mixed solution. When green turns reddish-brown, the high-temperature reaction ends. The high-temperature reaction ends when the green color changes to reddish-brown. At this time, 30 wt.% hydrogen peroxide solution was dropped into the mixed solution until no bubbles appeared. Finally, the obtained solution was sonicated for 30 min, and a higher purity GO solution was obtained after precipitation, centrifugation, and dialysis.

### 2.3. Preparation of Graphene-Cellulose-Polyethyleneimine Aerogel (GA-MCC-PEI)

First, 3 g of PEI solid was dissolved in 30 mL of deionized water and the mixture was stirred at a temperature of 80–100 °C for 2 h to obtain a completely dissolved 9 wt.% PEI aqueous solution. This process was repeated to prepare six groups of 9 wt.% PEI aqueous solution. Then 25 g/L GO solution was prepared by stirring 250 mg graphene oxide (GO) powder evenly in 10 mL deionized water. This process was repeated to prepare six groups of 25 g/L GO solution. Different amounts of cellulose powder (0 mg, 100 mg, 200 mg, 300 mg, 400 mg, and 500 mg) were added to each GO solution and the mixtures were stirred for 3 h to obtain a GO solution and graphene oxide-cellulose (GO-MCC) mixtures. A 25 mL PTFE-lined hydrothermal reactor was used to install these six groups of mixed solutions. All hydrothermal reactors were heated at 180 °C for 12 h to obtain graphene (GA) hydrogels and graphene-cellulose (GA-MCC) hydrogels. The six hydrogels were immersed in 9 wt.% PEI aqueous solution for 48 h to obtain a graphene-PEI (GA-PEI) hydrogel and graphene-cellulose-PEI (GA-MCC-PEI) hydrogels. In the process of freezing all hydrogels, first, use liquid nitrogen freezing, then, use a freeze dryer. GA-PEI aerogel and GA-MCC-PEI aerogel were obtained. Throughout the preparation process, the properties of all samples are not affected by other conditions to the maximum extent by strictly and accurately controlling the variables. The composite samples were named in order of increasing cellulose content: GA-PEI (MCC-0%), GA-MCC-PEI-1 (MCC-15.1%), GA-MCC-PEI-2 (MCC-25.5%), GA-MCC-PEI-3 (MCC-31.1%), GA-MCC-PEI-4 (MCC-38.9%), and GA-MCC-PEI-5 (MCC-43.1%). The contents of each component in the six groups of samples are shown in Table 1.

### 2.4. Characterization Method

The mass of the composite aerogel sample was weighed by an electronic balance. Scanning electron microscope (SEM) images were measured by QUANTA200. X-ray diffraction (XRD) measurements were performed using an XRD-6100 with a scan range of 5–60° and a scan speed of 5°/min. The specific surface area and pore structure of the composite aerogel were obtained by an ASAP2460 automatic specific surface area and porosity analyzer. For the compression test of the composite aerogel, the sample was cut into a cylindrical shape with a height of 0.5 cm and completed on a dynamic thermal analyzer model */DMA242E Artemis. Thermogravimetric analysis (TG) is a test performed using a 209 F3 Thermogravimetric Analyzer. A PE lambda 750 UV-Vis spectrophotometer was used to verify the adsorption properties of the composite aerogels for dyes. Fourier transform infrared spectroscopy (FTIR) was obtained using a Spectrum 400 instrument.

### 2.5. Adsorption Thermodynamic Equation

#### 2.5.1. Standard Curve Drawing of Amaranth and Methylene Blue

Deionized water was used as the reference solution. Seven different concentrations of the amaranth solution and methylene blue solution were set up, and the maximum absorbance was measured using an ultraviolet-visible (UV-vis) spectrophotometer at wavelengths of 552 nm and 664 nm for amaranth and methylene blue, respectively. Each experiment was repeated three times under the same conditions.

The general property parameters of dyes are shown in Table 2.

#### 2.5.2. Calculation of the Adsorption Capacity of the Sample to the Dye

The adsorption capacity of the sample for dyes is calculated as Formula (1) [26].

(1) $$q_{e}=\frac{\left(C_{0}{\;-\;C}_{e}\right)V}{W}$$

Formula: q_e_ is the equilibrium adsorption capacity (mg/g). C_0_ is the initial concentration of solvent (mg/L) before adsorption. C_e_ is the concentration of solvent (mg/L) when the adsorption equilibrium is reached. V is the volume of solvent (L). W is the quality of adsorbent (g).

#### 2.5.3. Adsorption Equilibrium Equation

In this paper, the Langmuir isothermal adsorption equation and the Freundlich isothermal adsorption equation were introduced, and the data on adsorption performance were analyzed using the curve-fitting method of these two equations.

Langmuir adsorption isotherm is an ideal monolayer adsorption model. The equation can be expressed as [29]:

(2) $$q_{e}=\frac{k_{L}C_{e}}{{1+k}_{L}C_{e}}Q_{m}$$

The equation can be transformed into:

(3) $$\frac{C_{e}}{q_{e}}=\frac{1}{k_{L}Q_{m}}+\frac{C_{e}}{Q_{m}}$$

Formula: q_e_ (mg/g) is the adsorption capacity of adsorbent at equilibrium. Concentration of C_e_ (mg/L) at equilibrium. k_L_ (L/mg) is the Langmuir isothermal adsorption constant, which describes the binding ability of the target to the adsorption site on the adsorbent. The greater the value, the greater the affinity between the adsorbate and the adsorbent is. Q_m_ (mg/g) is the saturated adsorption capacity of adsorbent.

Langmuir isothermal adsorption equation can also be used to describe the adsorption process by dimensionless R_L_, the expression is [29]:

(4) $$R_{L}=\frac{1}{{1+k}_{L}C_{0}}$$

Freundlich adsorption equation is a multi-molecular layer adsorption model. The equation can be expressed as [29]:

(5) $$Q_{e}{=K}_{F}C_{e}^{\frac{1}{n}}$$

Freundlich adsorption equation can be transformed into:

(6) $${\mathrm{logQ}}_{e}{=\mathrm{logK}}_{F}+\frac{1}{n}\cdot {\mathrm{logC}}_{e}$$

Formula: Q_e_ (mg/g) is the adsorption amount of adsorbent at equilibrium; concentration of Ce (mg/L) at equilibrium. K_F_ and 1/n are Freundlich isothermal equilibrium adsorption constants. The larger the K_F_ value, the larger the adsorption capacity. The larger the value of 1/n, the greater the adsorption strength. If 0.1 < 1/n < 0.5, this means that the adsorption process is spontaneous and easy.

## 3. Results

**Figure 1:**
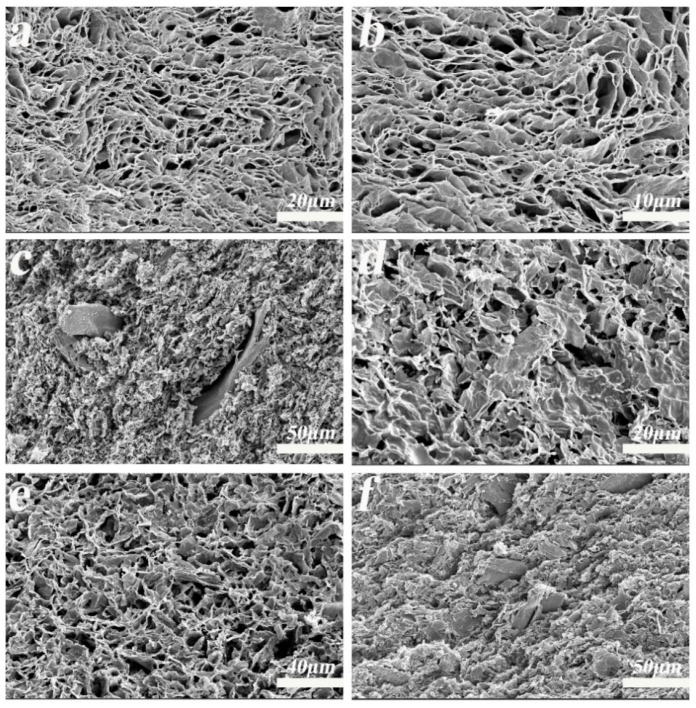
SEM images of GA-MCC-PEI aerogels: (**a**,**b**) GA-MCC-PEI-1, (**c**,**d**) GA-MCC-PEI-3, and (**e**,**f**) GA-MCC-PEI-5.

According to the SEM images of the GA-MCC-PEI aerogels, GA-MCC-PEI-(1, 3, 5) had irregular and densely packed pores. As shown in Figure 1a–f, rod-shaped cellulose was interspersed between the graphene sheets and formed a tightly packed structure with the graphene sheets. As shown in Figure 1c,f, the composite aerogel containing 43.11% cellulose (GA-MCC-PEI-5) had a greater number of pores and pores with thicker walls than the composite aerogel containing 31.12% cellulose (GA-MCC-PEI-3). The porous structure of the composite aerogels can provide sufficient transport channels for the diffusion of dyes when they are adsorbed to improve the adsorption efficiency of the adsorbent. Moreover, the tight connections between graphene and cellulose can enhance the mechanical properties of composite aerogels.

**Figure 2:**
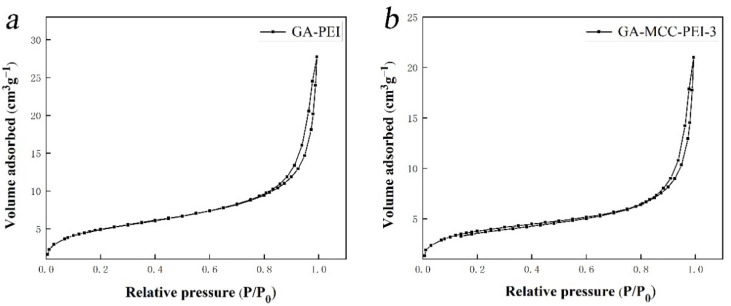
Brunauer–Emmett–Teller (BET) isotherms of (**a**) GA-PEI aerogel and (**b**) GA-MCC-PEI-3 aerogel for the determination of pore structure.

As shown in Figure 2, the Brunauer–Emmett–Teller (BET) isotherms of the GA-PEI and GA-MCC-PEI-3 aerogels both showed characteristics of type IV physical adsorption. Therefore, both the GA-PEI and GA-MCC-PEI-3 aerogels had typical mesoporous structures, which concurred with the dense porous structure revealed in the SEM images. The H3-type hysteresis regression line indicated that voids formed between the mesopores and wrinkled, wide, slit-like voids accumulated in the graphene layer, which induced nitrogen gas eruption during the desorption process [30]. A dense void structure is beneficial because it allows the composite aerogels to achieve the desired adsorption effect quickly and efficiently.

As shown in Table 3, both the GA-PEI and GA-MCC-PEI-(1–5) aerogels had large specific surface areas, and as the MCC content increased, the specific surface area of the aerogel first increased and then decreased. Among them, GA-MCC-PEI-4 (MCC-39%) had the largest specific surface area (92.32 m^2^/g). Because MCC is a filler with a high specific surface area and its linkages with GA and PEI increase the density of the composite aerogel, the specific surface area of the composite material gradually increases as the MCC content increases [31]. However, when the MCC content is too high (such as GA-MCC-PEI-5), the porous structure of the composite aerogel collapses, thus decreasing the specific surface area. In addition, when the density of the composite material is too large, the void volume in the aerogel may also reduce, thus slightly decreasing the specific surface area of the material. The specific surface area of GA-PEI (MCC-0%) was lower than that of GA-MCC-PEI-5 (MCC-43%), indicating that the linkages between cellulose and the other components, graphene and PEI, serve to optimize the porous structure of the composite aerogel. Moreover, the dense void structure enhances the adsorption capacity of the graphene-cellulose-PEI ternary composite aerogel.

**Figure 3:**
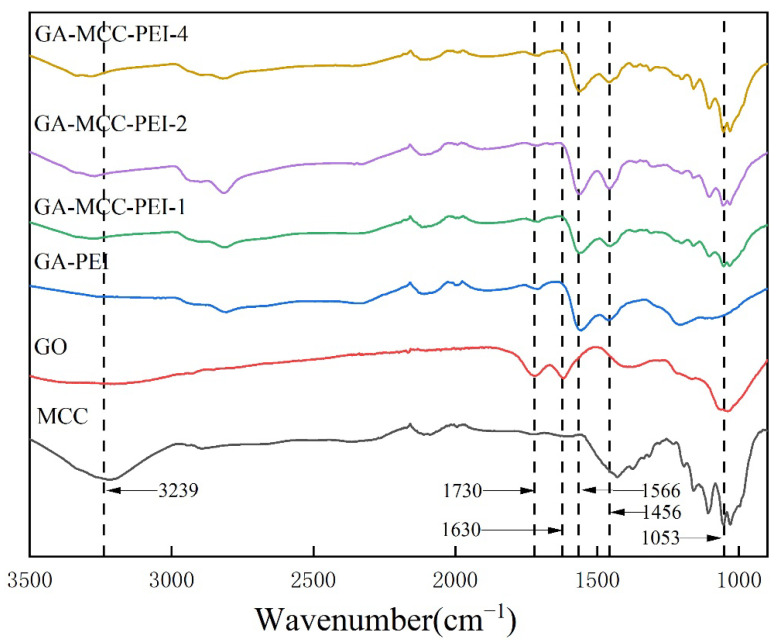
Infrared spectra of GO aerogel, MCC aerogel, GA-PEI aerogel, and GA-MCC-PEI-(1, 3, 5) aerogels.

As shown in Figure 3., the FTIR spectrum of the GO aerogel contained peaks at 1730 cm^−1^ and 1630 cm^−1^ corresponding to the stretching vibration of the C=O bond at the edge of GO and the bending vibration of the -COOH and -OH bonds, respectively [32]. These two peaks were significantly weakened or even disappeared in the FTIR spectra of the GA-PEI and GA-MCC-PEI-(1, 3, 5) aerogels. Moreover, a peak (1566 cm^−1^) corresponding to the stretching vibration of the C=C bond of GA appeared in the composite aerogel, which indicated that GO was reduced to GA during the preparation of the composite aerogel [33]. The FTIR spectra of the MCC and GA-MCC-PEI-(1, 3, 5) aerogels contained peaks corresponding to the O-H vibrational contraction (3239 cm^−1^) and C-H vibrational contraction (1456 cm^−1^). These characteristic peaks of cellulose indicated that MCC was incorporated into the GA-MCC-PEI aerogels [34]. With the increase in MCC content, the O-H peak (3200–3500 cm^−1^) and C-H peak (1456 cm^−1^) shifted, indicating that the O-H and C-H groups in the composite aerogel were connected to form hydrogen bonds. In the spectra of both the GA-PEI and GA-MCC-PEI aerogels, the peak at 1566 cm^−1^ corresponding to the bending vibration of the -NH bond of the secondary amine indicated that PEI was successfully integrated into the composite aerogels [35].

**Figure 4:**
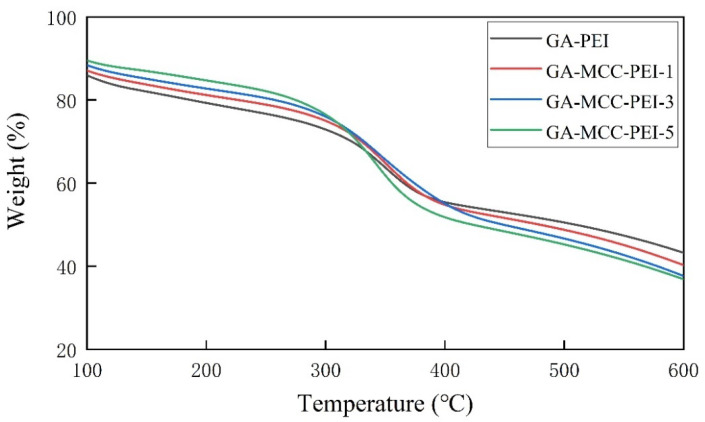
Thermogravimetric analysis of the GA-PEI aerogel and GA-MCC-PEI-(1, 3, 5) aerogels.

Thermogravimetric analyses (Figure 4) of the GA-PEI and GA-MCC-PEI-(1, 3, 5) aerogels revealed mass losses. The mass loss occurred in the temperature range of 230–450 °C and was insignificant at temperatures below 200 °C. For all samples, the mass loss occurred during three main stages. When the temperature is 0~200 °C, the weight loss of all composite aerogel samples is less than 15%, which is the first stage of thermogravimetric, indicating that residual water in the pores of the aerogel was removed, whereas the sample itself did not suffer a mass loss. This result concurred with the SEM and BET results in that the composites contain a dense pore structure. When the temperature is 200–420 °C, all the composite aerogels have a significant mass loss, which is the second stage of thermogravimetric. The mass loss ranged from 24% to 38%, suggesting pyrolysis of cellulose and decomposition of hydrogen bonds [36]. The third stage occurred at 450–700 °C, and the mass loss ranged from 25% to 35%, indicating that the residual carbon was oxidatively degraded into gaseous substances with low molecular weights [37].

From a comparison of the thermogravimetric curves of the GA-PEI and GA-MCC-PEI-(1, 3, 5) aerogels (Figure 4), as the MCC content increased, the thermal performance of the composite material increased. The stability first increased and then decreased, indicating that the hydrogen bond can enhance the thermal stability of composite aerogels. However, when the MCC content was high (GA-MCC-PEI-5), pyrolysis of a large amount of MCC reduced the thermal stability of the sample. The thermogravimetric loss of 30 wt.% occurred at 322.2 °C for the GA-PEI aerogel, 330.4 °C for GA-MCC-PEI-1, 332.4 °C for GA-MCC-PEI-3, and 326.8 °C for GA-MCC-PEI-5. These temperatures were higher than that (224 °C) for the cotton stalk bark MCC-GO aerogel prepared by Li et al. [38]. In general, the thermal stability of the composite aerogel can be maximized by controlling the MCC content, and good thermal stability can widen the application space of composite aerogels in the field of adsorption.

**Figure 5:**
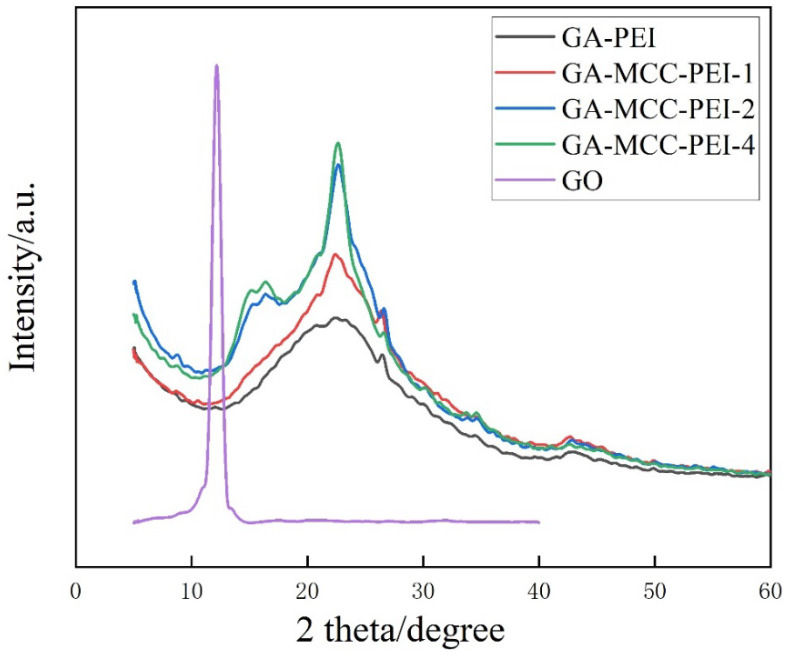
X-ray diffraction patterns of GO aerogel, GA-PEI aerogel, and GA-MCC-PEI-(1, 2, 4) aerogels.

As shown in Figure 5, there was only one peak (11.6°) in the XRD pattern of the GO aerogel, which corresponded to the (002) plane of GO [39]. According to the Bragg equation, the interlayer spacing (d) was 0.76 nm. In the XRD patterns of the GA-PEI and GA-MCC-PEI-(1, 2, 4) aerogels, there were no characteristic peaks of GO. A peak at 22.4° corresponding to the characteristic peak of GA was observed [40]. The calculation showed that its interlayer spacing was 0.39 nm. Therefore, GO was completely reduced to GA, which agreed with the FTIR results. A characteristic peak (16.5°) of MCC appeared in the XRD pattern of the GA-MCC-PEI-(1, 2, 4) aerogels, corresponding to the 10Ῑ facet of MCC [41]. As the MCC content increased, the characteristic peak of MCC increased in intensity and the graphene peak shifted to the left. Therefore, a large number of oxygen-containing functional groups was removed during the reduction of GO, and graphene sheets were re-stacked under the action of π–π interactions, despite MCC inserted between graphene sheets reducing the stacking effect of π–π interactions.

**Figure 6:**
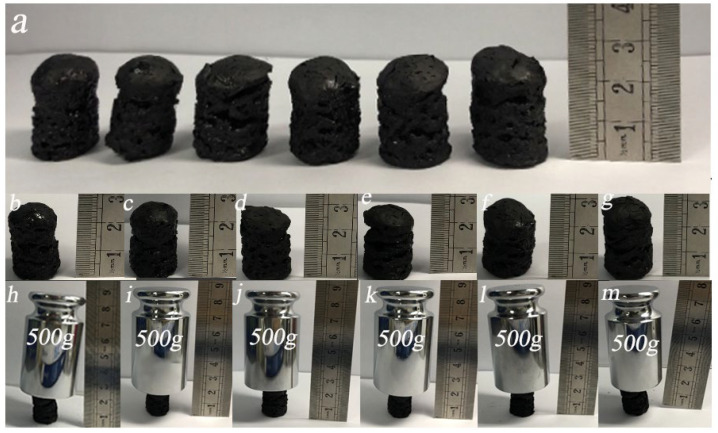
Compression testing of the GA-PEI and GA-MCC-PEI-(1–5) aerogels: (**a**) photograph of all samples, (**b**–**g**) photographs of individual samples, and (**h**–**m**) photographs of samples under 500 g pressure.

As shown in Figure 6a, the GA-PEI aerogel and GA-MCC-PEI-(1–5) aerogels all had a cylindrical structure (radius of 1.1 cm and height of 2.48–2.86 cm) and a porous surface before compression testing. Height maps of the GA-PEI aerogel and GA-MCC-PEI-(1–5) aerogels are shown in Figure 6b–g. When the MCC content in composite aerogels increases gradually, their height also increases. As shown in Figure 6h–m, when the GA-PEI and GA-MCC-PEI-(1–5) aerogels were subjected to a pressure of 500 g (approximately 553 times their own weight), the composite aerogel did not break or crush and the height did not change significantly. Therefore, the GA-PEI and GA-MCC-PEI aerogels exhibited excellent anti-compression properties.

**Figure 7:**
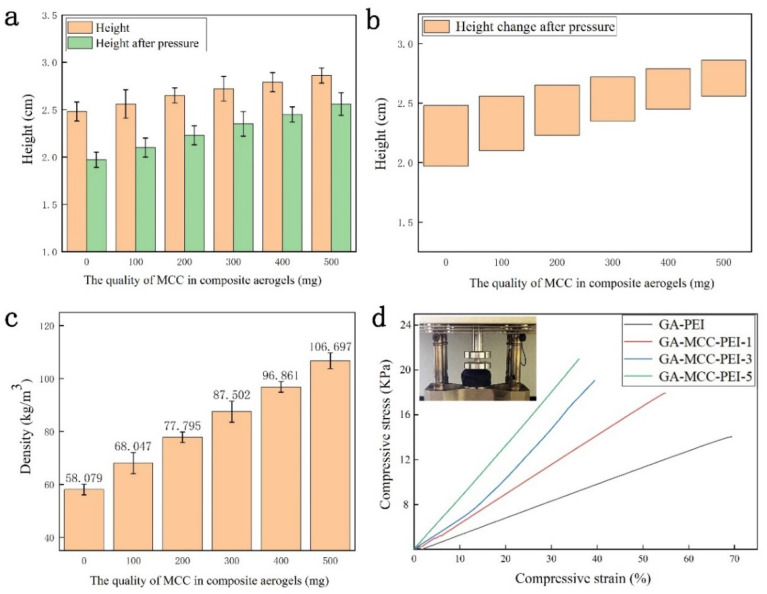
(**a**) Height maps of the GA-PEI and GA-MCC-PEI-(1–5) aerogels, (**b**) height change map after pressure, (**c**) density numerical map, and (**d**) stress—strain curve.

Due to the porosity of aerogels, there is more demand for improving their mechanical properties. As shown in Figure 7a,b, the height of the GA-PEI and GA-MCC-PEI-(1–5) aerogels changed when they were subjected to a pressure of 500 g (approximately 553 times their own weight). Moreover, as the MCC content increased, the height change of the composite aerogel decreased. Among them, GA-MCC-PEI-5 (MCC content of 43%) exhibited the smallest change in height under compression. Therefore, the addition of MCC enhances the mechanical properties of the composite by promoting the formation of hydrogen bonds among PEI, GA, and MCC, as supported by the XRD and FTIR results. As shown in Figure 7c,d, at low MCC contents, the density of the composite aerogel was low and the internal pores were large, and thus the deformation was high. As the MCC content increased, the density of the composite aerogel increased and the size of the pores decreased. The tighter the arrangement of the aerogel, the greater the pressure it can withstand. This also shows that the hydrogen bonding among MCC, GA, and PEI can enhance the mechanical properties of the composites. As shown in Figure 6d, GA-MCC-PEI-5 (MCC-43%) with the highest MCC content had the largest Young’s modulus and was able to withstand a maximum stress of 21.76 kPa, approximately 827 times its weight.

Table 4 shows the maximum allowable pressure of composite aerogels prepared from graphene, cellulose, and PEI reported in recent years. These values were lower than those (21.76 kPa) of GA-MCC-PEI-5 (MCC-43%), which was attributed to the hydrogen bonding between graphene, cellulose, and PEI. This shows that for porous aerogel materials, by reasonably adjusting the content of MCC, GA, and PEI to control the number of hydrogen bonds in the composite material, a composite aerogel with ideal mechanical properties can be obtained.

**Figure 8:**
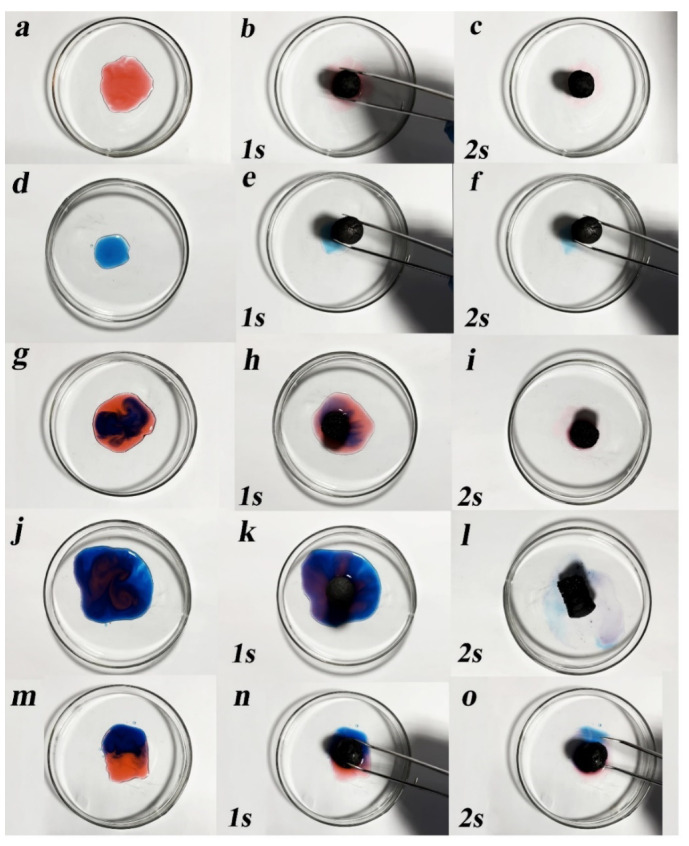
Adsorption capacity of the GA-MCC-PEI-4 aerogel for (**a**–**c**) amaranth, (**d**–**f**) methylene blue, (**g**–**i**) mixture of amaranth and methylene blue (more amaranth), (**j**–**l**) mixture of amaranth and methylene blue (more methylene blue), and (**m**–**o**) mixture of equal amounts of amaranth and methylene blue within 1 s and 2 s.

The SEM and BET results show that the composite aerogel has a dense mesoporous structure and can be applied to the adsorption of dyes in water. Therefore, the GA-MCC-PEI-4 aerogel (MCC-39%) with the largest specific surface area among the composite aerogels was selected for the adsorption tests. As shown in Figure 8a–f, droplets of amaranth and methylene blue (0.1 g/L solutions) were adsorbed by GA-MCC-PEI-4 within 2 s. The traces of dye remaining in the petri dishes indicated that GA-MCC-PEI-4 had a greater adsorption capacity for amaranth than for methylene blue. In particular, compared with the methylene blue traces, there were fewer amaranth traces and they were lighter in color. Dripping of methylene blue into amaranth (Figure 8g–i), dripping of amaranth into methylene blue (Figure 8j–l), and adjacent dripping of the two dyes (Figure 8m,n) revealed that the dry GA-MCC-PEI-4 aerogel adsorbed the dyes within 2 s. A comparison of Figure 8i,l also indicated that GA-MCC-PEI-4 had a greater adsorption capacity for amaranth. In particular, compared with the methylene blue traces, there were fewer amaranth traces and they were lighter in color after the adsorption time of 2 s. Similarly, as shown in Figure 8m–o, GA-MCC-PEI-4 had a greater adsorption capacity for amaranth than for methylene blue. Amaranth and methylene blue solutions (same concentrations) were dropped adjacently into a petri dish with GA-MCC-PEI-4 placed in the middle for adsorption. After 2 s (Figure 8o), it was clear that the composite aerogel had a stronger adsorption capacity for amaranth than for methylene blue.

In summary, the GA-MCC-PEI-4 composite aerogel had obvious adsorption capacity for cationic dye methylene blue and anionic dye amaranth, but it had a better adsorption effect on amaranth. The porous structure of the composite aerogel is the reason for the adsorption of these two dyes, so it has a physical adsorption effect on amaranth dye and methylene blue dye. Furthermore, GA-MCC-PEI-4 composite aerogels (PEI-12.4%) contain higher cationic PEI and have a stronger and more sensitive affinity to the anionic dye amaranth. When composite aerogels were used to adsorb the amaranth dye, chemical adsorption also occurred. In short, the adsorption process of GA-MCC-PEI on the cationic dye methylene blue is mainly the physical adsorption process caused by the interaction between the adsorbate and the adsorbent molecule, and its adsorption on the anionic dye amaranth occurs as both physical and chemical adsorption. Chemical adsorption is the adsorption of electron transfer, exchange, or common between adsorbate molecules and atoms (or molecules) on the solid surface to form adsorption chemical bonds. This is also why when composite aerogels make contact with the cationic dye methylene blue and the anionic dye amaranth at the same time, they tend to adsorb more amaranth dyes before reaching their maximum adsorption capacity.

**Figure 9:**
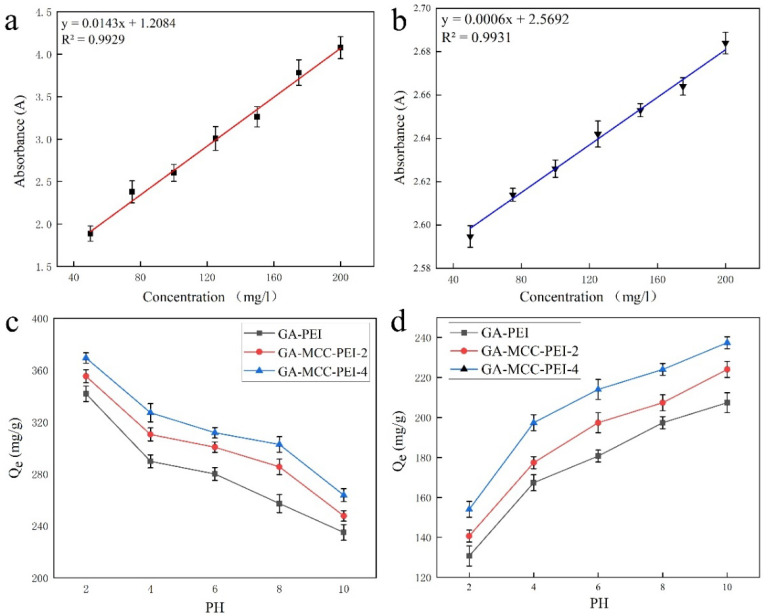
Standard curves of (**a**) amaranth and (**b**) methylene blue. Adsorption capacity curves of (**c**, **d**) GA-PEI, GA-MCC-PEI-2, and GA-MCC-PEI-4 under different pH conditions. Equilibrium absorption capacities for amaranth and methylene blue.

As shown in Figure 9a,b, as the concentrations of amaranth and methylene blue increased, the corresponding absorbance values also increased. The fitted standard curves of absorbance versus concentration for the two dyes had correlation coefficient (R^2^) values that were greater than 0.99. Therefore, these standard curves were used in subsequent concentration calculations.

As shown in Figure 9c,d, the adsorption capacities of GA-PEI (MCC-0%), GA-MCC-PEI-2 (MCC-26%), and GA-MCC-PEI-4 (MCC-39%) for amaranth and methylene blue were significantly affected by the pH value. In particular, the adsorption capacities of the aerogels for the anionic dye amaranth decreased as the pH increased, whereas the adsorption capacities of the aerogels for the cationic dye methylene blue increased as the pH increased. When GA-MCC-PEI adsorbs the anionic dye amaranth, the adsorption amount of amaranth dye can be divided into three parts with the change in PH. A PH between 2 and 4 is a high adsorption area, a PH between 4 and 8 is a transition area, and a PH between 8 and 10 is a low adsorption area. When GA-MCC-PEI adsorbs methylene blue, the adsorption amount of methylene blue dye can also be divided into three parts with the change in PH. A PH between 2 and 4 is a low adsorption area, a PH between 4 and 8 is a transition area, and a PH between 8 and 10 is a high adsorption area. In low-pH environments, excessive H^+^ inhibits the release of cations, resulting in the weak adsorption capacity of the aerogels for the cationic dye methylene blue. In contrast, in low-pH environments, excessive H^+^ promotes the release of anions and thus the adsorption of anionic dyes (amaranth). Therefore, as the pH increased, the adsorption capacity of the aerogel changed in opposite directions for anionic dyes (amaranth) and cationic dyes (methylene blue).

As shown in Figure 9c,d, the equilibrium adsorption capacity of the aerogel for the dyes increased as the MCC content increased. As the MCC content increased, the pores in the composite aerogel increased in number and decreased in size, thus increasing the adsorption capacity. In addition, the equilibrium adsorption capacity of the composite aerogel was higher for anionic dyes (amaranth) than for cationic dyes (methylene blue) irrespective of the MCC content and the pH environment because of the high affinity of cationic PEI in the composite aerogel for anionic dyes, which also confirmed the results shown in Figure 8g–o. GA-MCC-PEI-4 (MCC-39%) had the maximum adsorption capacity for both dyes, with an equilibrium adsorption amount of 369.37 mg/g (pH = 2) for amaranth and 237.33 mg/g (pH = 10) for methylene blue.

Table 5 shows the maximum adsorption capacities of composites prepared from cellulose, graphene, and PEI for amaranth dye and methylene blue dye in recent years, which are all lower than that of GA-MCC-PEI-4 (MCC-39%) for amaranth (369.37 mg/g) and methylene blue (237.33 mg/g). Therefore, the adsorption capacity of a composite aerogel can be improved by regulating the content of cellulose in the composite to optimize the porous structure of the aerogel.

**Figure 10:**
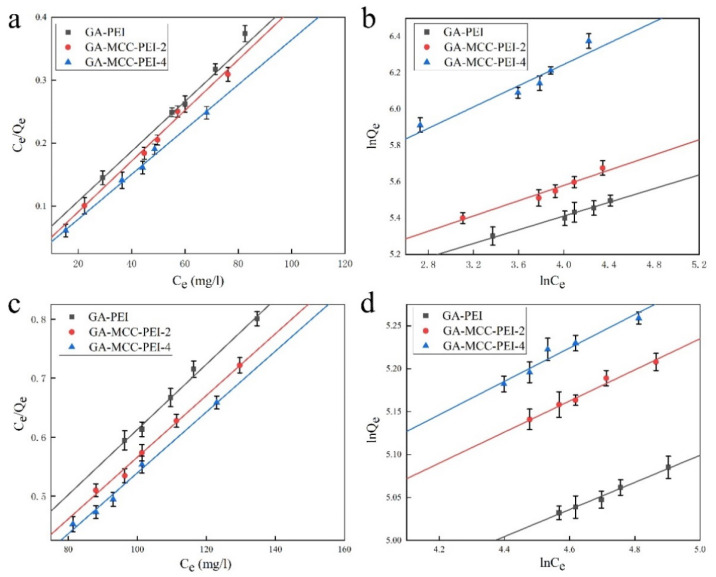
Fitting of adsorption isotherms: Langmuir (**a**,**c**) and Freundlich (**b**,**d**) isotherms for the adsorption of amaranth (**a**,**b**) and methylene blue (**c**,**d**) by GA-PEI, GA-MCC-PEI-2, and GA-MCC-PEI-4.

As shown in Figure 10, the adsorption curves of GA-PEI (MCC-0%), GA-MCC-PEI-2 (MCC-26%), and GA-MCC-PEI-4 (MCC-39%) were fitted to the Langmuir and Freundlich isothermal equations with good R^2^ values. Among them, the initial concentrations of the composite aerogels for the adsorption of the two dyes were determined by pre-experiment. It is the initial concentration corresponding to the maximum absorption of composite aerogels under certain conditions (pH = 7, room temperature).

As shown in Table 6, the Freundlich isothermal equation showed good fitting with the experimental data (R^2^ > 0.9). The Freundlich isotherm is used to describe adsorption processes that occur on heterogeneous surfaces and active sites with different energies based on multilayer adsorption and equilibrium [60]. Adsorption strength n is used to describe the inhomogeneity of the adsorption surface [61]. The greater the value of 1/n, the greater the adsorption strength of the adsorbate. When 0.1 < 1/n < 0.5, the adsorption process is able to proceed. K_F_ describes the adsorption capacity of the adsorption process. The larger the value, the larger the adsorption capacity of the adsorbate [62,63]. The 1/n of the composite aerogel GA-MCC-PEI for the adsorption of the amaranth or methylene blue solutions was between 0.1 and 0.5. Moreover, with the increase in the MCC content in the composite aerogels, the values of 1/n and KF gradually increased. For the composite aerogels with the same MCC content, the values of 1/n and K_F_ corresponding to the absorption of the amaranth dye are higher than those corresponding to the absorption of the methylene blue dye. This indicates that the adsorption process of the GA-MCC-PEI composite aerogel for the two dyes is beneficial. Moreover, with the increase in the MCC content in the composite aerogel, its absorption capacity is stronger. The absorption capacity of the GA-MCC-PEI composite aerogel for the amaranth dye was better than that for the methylene blue dye.

The Langmuir equation showed a good fitting with the experimental data (R^2^ > 0.99), which was greater than the corresponding R^2^ value fitted by the Freundlich isothermal equation. Therefore, the Langmuir adsorption model described the adsorption isotherms better than the Freundlich adsorption model, indicating that monolayer adsorption of the two dyes occurred in all the composite aerogel samples. The dimensionless R_L_ values of the Langmuir equation (Table 6) were greater than 0 and less than 1, indicating that all the adsorption processes were spontaneous. Moreover, as the MCC content increased, both the isothermal adsorption constant k_L_ and saturated adsorption capacity Q_m_ of the composite aerogel increased. This shows that with the increase in the MCC content in the composite aerogel, its absorption capacity is stronger. The GA-MCC-PEI-4 (MCC-39%) aerogel with the largest specific surface area had the best adsorption capacity because the density of the pores in the composite aerogel was optimal at an MCC content of 39%. For the composite aerogels with the same MCC content, the isothermal adsorption constant k_L_ and saturated adsorption capacity Q_m_ corresponding to the amaranth dye absorption were higher than those corresponding to the methylene blue dye absorption. All samples showed a greater adsorption capacity for the anionic dye amaranth than for the cationic dye methylene blue because of the high affinity of cationic PEI in the composite aerogel for anionic dyes.

## 4. Conclusions

In this work, a preparation method for ternary composite aerogels composed of graphene, cellulose, and PEI was demonstrated. Ultra-lightweight graphene-cellulose-PEI aerogels (GA-MCC-PEI) with a dense mesoporous structure on the surface and with high mechanical strength, good thermal stability, and significant adsorption capacity for both anionic and cationic dyes were prepared. Inside the composite aerogel, hydrogen bonds formed among cellulose, graphene, and PEI. Cellulose was successfully inserted between the graphene sheets, which played a role in preventing the π–π stacking of the graphene sheets. The GA-MCC-PEI aerogel with an MCC content of 43% (mass of 0.91 g) was able to withstand a pressure of up to 21.76 kPa, equivalent to 827 times the mass of the aerogel. As the cellulose content increased, the mechanical properties of the composite aerogel were enhanced. Adsorption tests revealed that the GA-MCC-PEI composite aerogel had a significant adsorption capacity for the anionic dye amaranth and the cationic dye methylene blue. Moreover, fitting of the adsorption isotherms using the Langmuir isothermal equation yielded high correlation coefficients, indicating monolayer adsorption. Among the prepared aerogels, GA-MCC-PEI-4 (MCC-39%) had the largest adsorption capacity for both dyes, with an equilibrium adsorption capacity of 369.37 mg/g and 237.33 mg/g for amaranth and methylene blue, respectively. The prepared graphene-cellulose-PEI aerogel can be used to remove dye pollutants in water to maintain ecological balance, thus broadening the application space of aerogel materials, that is, as adsorbents in different environments.

## Figures and Tables

**Table 1:** Content of each component in composite aerogels.

Composite Aerogel	GA (wt.%)	MCC (wt.%)	PEI (wt.%)	MCC:GA:PVA
GA-PEI	91.4	0	8.6	0:5:0.5
GA-MCC-PEI-1	75.5	15.1	9.3	1:5:0.6
GA-MCC-PEI-2	63.8	25.5	10.6	2:5:0.8
GA-MCC-PEI-3	55.3	31.1	11.5	2.8:5:1
GA-MCC-PEI-4	48.7	38.9	12.4	4:5:1.3
GA-MCC-PEI-5	43.1	43.1	13.8	5:5:1.6

**Table 2:** Conventional property parameters of dyes.

Dyestuff Type	Chemotaxonomy	Molecular Formula	Molecular Structure	λ Max (nm)
Amaranth red (AM)	Anionic dye	604.5	C_20_H_11_N_2_Na_3_O_10_S_3_	552
Methylene blue (MB)	Cationic dye	373.9	C_16_H_18_ClN_3_S·3H_2_O	664

**Table 3:** Specific surface areas of GA aerogel and GA-MCC-PEI-(1–5) aerogel.

MCC Content in the Sample (mg)	0	100	200	300	400	500
Specific surface area (m^2^/g)	84.19	85.28	87.77	89.14	92.32	88.35

**Table 4:** Maximum allowable pressures of MCC composite aerogel, GA composite aerogel, and PEI composite aerogel.

Composite Aerogel	Maximum Withstand Pressure (kPa)	Reference
Cellulose-graphene aerogel	6.0	[19]
Polyvinyl alcohol/cellulose nanocrystal/graphene composite aerogels	4.0	[42]
Fe_3_O_4_/cellulose/polyvinyl alcohol hybride aerogel	6.5	[43]
NiO-Fe_2_O_3_/graphene/polyvinyl alcohol aerogel	9.0	[44]
Graphene/cellulose nanocrystals hybrid aerogel	8.9	[45]
CaCO_3_-decorated cellulose aerogel	4.5	[46]
Cellulose/polyethyleneimine aerogel	10.5	[47]
Cellulose nanofibril/emulsion composite aerogel	18.2	[48]
Cellulose nanofibrils/APMDS aerogel	9.75	[49]
Carbon aerogel from cellulose nanofibers	7	[50]
Graphene oxide/cellulose-derived carbon aerogel	18	[51]
Graphene-cellulose-polyethyleneimine aerogel	21.76	This work ^1^

^1^: The graphene-cellulose-polyethyleneimine aerogel (MCC-43%) prepared in this experiment.

**Table 5:** Maximum absorption capacities of MCC composites, GA composites, and PEI composites as adsorbents for amaranth or methylene blue.

Composite Material	Absorption of AM (mg/g)	Absorption of MB (mg/g)	Reference
Graphene/waste-newspaper cellulose composite aerogels		154	[52]
N, N′-methylene bisacrylamide-cellulose sponge		123.46	[53]
Polyvinyl alcohol/carboxymethyl cellulose hydrogels		172.14	[54]
Cellulose nanofiber aerogel		196.08	[55]
Cellulose/diatomite composite aerogel spheres		71.94	[56]
Polydopamine/polyethyleneimine/graphene oxide aerogel	196.7		[57]
Quaternary ammonium base cellulose	360		[58]
Fe_3_O_4_/mZrO_2_/reduced graphite oxide	76.9		[59]
Graphene-cellulose-polyethyleneimine aerogel	369.37	237.33	This work

**Table 6:** Isotherm parameters for the adsorption of amaranth and methylene blue by GA-PEI, GA-MCC-PEI-2, and GA-MCC-PEI-4.

**Dyestuff Type**	Sample Types	Langmuir Model				Freundlich Model		
		K_L_	Q_m_	R^2^	R_L_	K_F_	1/n	R^2^
		(L/mg)	(mg/g)			(mg/g)		
AM	GA-PEI	0.25	338.09	0.994	0.02	207.89	0.182	0.9839
	GA-MCC-PEI-2	0.33	350.32	0.991	0.015	211.44	0.216	0.9651
	GA-MCC-PEI-4	0.39	377.78	0.993	0.013	261.58	0.291	0.9262
MB	GA-PEI	0.09	281.82	0.992	0.053	173.4	0.160	0.9896
	GA-MCC-PEI-2	0.13	288.68	0.993	0.037	177.25	0.177	0.9824
	GA-MCC-PEI-4	0.19	296.01	0.991	0.026	179.62	0.184	0.9455

## Data Availability

The data that support the findings of this study are available from the corresponding author upon reasonable request.

## References

[1] Nguyen V.T., Ha L.Q., Nguyen T.D.L., Ly P.H., Nguyen D.M., Hoang D. (2022). Nanocellulose and graphene oxide aerogels for adsorption and removal methylene blue from an aqueous environment. ACS Omega.

[2] Massaro M., Campisciano V., Iborra C.V., Liotta L.F., Sanchez-Polo M., Riela S., Gruttadauria M. (2020). New mussel inspired polydopamine-like silica-based material for dye adsorption. Nanomaterials.

[3] Li S.S., Zhang H.J., Hu S.Y., Liu J., Zhu Q., Zhang S.W. (2019). Synthesis of hierarchical porous carbon in molten salt and its application for dye adsorption. Nanomaterials.

[4] Wang C.C., Yang S.D., Ma Q., Jia X., Ma P.C. (2017). Preparation of carbon nanotubes/graphene hybrid aerogel and its application for the adsorption of organic compounds. Carbon.

[5] Pruna A., Carcel A.C., Barjola A., Benedito A., Gimenez E. (2019). Tailoring the performance of graphene aerogels for oil/organic solvent separation by 1-step solvothermal approach. Nanomaterials.

[6] Li M.Z., Miao Y.Y., Zhai X.Y., Yin Y.X., Zhang Y.T., Jian Z.B., Wang X.Y., Sun L.P., Liu Z.B. (2019). Preparation of and research on bioinspired graphene oxide/nanocellulose/polydopamine ternary artificial nacre. Mater. Des..

[7] Zhi D.D., Li T., Li J.Z., Ren H.S., Meng F.B. (2021). A review of three-dimensional graphene-based aerogels: Synthesis, structure and application for microwave absorption. Compos. B Eng..

[8] Lin X., Li Y.J., Chen F.T., Xu P., Li M. (2017). Facile synthesis of mesoporous titanium dioxide doped by Ag-coated graphene with enhanced visible-light photocatalytic performance for methylene blue degradation. RSC Adv..

[9] Chen H., Chen Z.H., Yang H., Wen L.H., Yi Z., Zhou Z.G., Dai B., Zhang J.G., Wu X.W., Wu P.H. (2022). Multi-mode surface plasmon resonance absorber based on dart-type single-layer graphene. RSC Adv..

[10] Tang N.M., Li Y.J., Chen F.T., Han Z.Y. (2018). In situ fabrication of a direct Z-scheme photocatalyst by immobilizing CdS quantum dots in the channels of graphene-hybridized and supported mesoporous titanium nanocrystals for high photocatalytic performance under visible light. RSC Adv..

[11] Stankovich S., Dikin D.A., Piner R.D., Kohlhaas K.A., Kleinhammes A., Jia Y., Wu Y., Nguyen S.T., Ruoff R.S. (2007). Synthesis of graphene-based nanosheets via chemical reduction of exfoliated graphite oxide. Carbon.

[12] Liu H.M., Qiu H.D. (2020). Recent advances of 3D graphene-based adsorbents for sample preparation of water pollutants: A review. Chem. Eng. J..

[13] Li M.Z., Wang X.Y., Zhao R., Miao Y.Y., Liu Z.B. (2021). A novel graphene-based micro/nano architecture with high strength and conductivity inspired by multiple creatures. Sci. Rep..

[14] Miao Y.Y., Zhang C.Y., Huang D.J., Tian L., Zhao T.J., Zhai X.Y., Liu Z.B. (2018). Preparation of graphene oxide /cellulose composite in mixed acid solution derived from Hummers method. J. For. Eng..

[15] Huang B., Lin F.C., Tang L.R., Lu Q.L., Lu B.L. (2022). Research advances of functional cellulose-based hydrogels and its applications. J. For. Eng..

[16] Wang X.Y., Wan K., Xie P.B., Miao Y.Y., Liu Z.B. (2021). Ultralight, high capacitance, mechanically strong graphene-cellulose aerogels. Molecules.

[17] Zheng Q.F., Cai Z.Y., Ma Z.Q., Gong S.Q. (2015). Cellulose nanofibril/reduced graphene oxide/carbon nanotube hybrid aerogels for highly flexible and all-solid-state supercapacitors. ACS Appl. Mater. Interfaces.

[18] Jiang Q.S., Kacica C., Soundappan T., Liu K.K., Tadepalli S., Biswas P., Singamaneni S. (2017). An in situ grown bacterial nanocellulose/graphene oxide composite for flexible supercapacitors. J. Mater. Chem. A.

[19] Zhao L., Wang Z.B., Li J.L., Zhang J.J., Sui X.L., Zhang L.M. (2015). One-pot synthesis of a three-dimensional graphene aerogel supported Pt catalyst for methanol electrooxidation. RSC Adv..

[20] Joshi P., Sharma O.P., Ganguly S.K., Srivastava M., Khatri O.P. (2022). Fruit waste-derived cellulose and graphene-based aerogels: Plausible adsorption pathways for fast and efficient removal of organic dyes. J. Colloid Interface Sci..

[21] Tangtubtim S., Saikrasun S. (2019). Adsorption behavior of polyethyleneimine-carbamate linked pineapple leaf fiber for Cr(VI) removal. Appl. Surf. Sci..

[22] Truong H.B., Ike I.A., Ok Y.S., Hur J. (2020). Polyethyleneimine modification of activated fly ash and biochar for enhanced removal of natural organic matter from water via adsorption. Chemosphere.

[23] Kani A.N., Dovi E., Mpatani F.M., Aryee A.A., Han R.P., Li Z.H., Qu L.B. (2022). Pollutant decontamination by polyethyleneimine-engineered agricultural waste materials: A review. Environ. Chem. Lett..

[24] Choi W., Min K., Kim C., Ko Y.S., Jeon J., Seo H., Park Y.K., Choi M. (2016). Epoxide-functionalization of polyethyleneimine for synthesis of stable carbon dioxide adsorbent in temperature swing adsorption. Nat. Commun..

[25] Li J., Zuo K.M., Wu W.B., Xu Z.Y., Yi Y.G., Jing Y., Dai H.Q., Fang G.G. (2018). Shape memory aerogels from nanocellulose and polyethyleneimine as a novel adsorbent for removal of Cu(II) and Pb(II). Carbohydr. Polym..

[26] Wang C., Okubayashi S. (2019). Polyethyleneimine-crosslinked cellulose aerogel for combustion CO_2_ capture. Carbohydr. Polym..

[27] Fang Y.Y., He H., Dong K.Q., Yang J.S., Qin Z.Y. (2022). Preparation and adsorption properties of hyperbranched polyethyleneimine-cellulose nanofiber aerogel. New J. Chem..

[28] Miao Y.Y., Wang X.Y., Liu Y.X., Liu Z.B., Chen W.S. (2021). Preparation of graphene oxide/cellulose composites with microcrystalline cellulose acid hydrolysis using the waste acids generated by the hummers method of graphene oxide synthesis. Polymers.

[29] Kadirvelu K., Goel J., Rajagopal C. (2008). Sorption of lead, mercury and cadmium ions in multi-component system using carbon aerogel as adsorbent. J. Hazard. Mater..

[30] Wang X.Y., Xie P.B., Wan K., Miao Y.Y., Liu Z.B., Li X.J., Wang C.X. (2021). Mechanically strong, low thermal conductivity and improved thermal stability polyvinyl alcohol-graphene-nanocellulose aerogel. Gels.

[31] Zhao S.Y., Zhang Z., Sebe G., Wu R., Virtudazo R.V.R., Tingaut P., Koebel M.M. (2015). Multiscale assembly of superinsulating silica aerogels within silylated nanocellulosic scaffolds: Improved mechanical properties promoted by nanoscale chemical compatibilization. Adv. Funct. Mater..

[32] Kang Y.J., Chun S.J., Lee S.S., Kim B.Y., Kim J.H., Chung H., Lee S.Y., Kim W. (2012). All-solid-state flexible supercapacitors fabricated with bacterial nanocellulose papers, carbon nanotubes, and triblock-copolymer ion gels. ACS Nano.

[33] Tucureanu V., Matei A., Avram A.M. (2016). FTIR spectroscopy for carbon family study. Crit. Rev. Anal. Chem..

[34] Yang H.P., Yan R., Chen H.P., Lee D.H., Zheng C.G. (2007). Characteristics of hemicellulose, cellulose and lignin pyrolysis. Fuel.

[35] Xie X.Q., Wang Y.F., Xiong Z., Li H.Z., Yao C. (2020). Highly efficient removal of uranium(VI) from aqueous solution using poly(cyclotriphosphazene-co-polyethyleneimine) microspheres. J. Radioanal. Nucl. Chem..

[36] Zhou T., Cheng X.D., Pan Y.L., Li C.C., Gong L.L. (2019). Mechanical performance and thermal stability of polyvinyl alcohol-cellulose aerogels by freeze drying. Cellulose.

[37] Yang J., Zhang E.W., Li X.F., Zhang Y.T., Qu J., Yu Z.Z. (2016). Cellulose/graphene aerogel supported phase change composites with high thermal conductivity and good shape stability for thermal energy storage. Carbon.

[38] Li Z.Q., Ma X.D., Wei C.Y., Zheng L.J., Yan J., Zheng H.D. (2020). Effect of graphene oxide on the structure of cotton stalk bast cellulose aerogel. Fine Chem..

[39] Stobinski L., Lesiak B., Malolepszy A., Mazurkiewicz M., Mierzwa B., Zemek J., Jiricek P., Bieloshapka I. (2014). Graphene oxide and reduced graphene oxide studied by the XRD, TEM and electron spectroscopy methods. J. Electron. Spectrosc. Relat. Phenom..

[40] Andonovic B., Ademi A., Grozdanov A., Paunovic P., Dimitrov A.T. (2015). Enhanced model for determining the number of graphene layers and their distribution from X-ray diffraction data. Beilstein J. Nanotechnol..

[41] Yao W.Q., Weng Y.Y., Catchmark J.M. (2020). Improved cellulose X-ray diffraction analysis using Fourier series modeling. Cellulose.

[42] Wu Y., Wu X.Y., Yang F., Xu L., Sun M. (2019). Study on the preparation and adsorption property of polyvinyl alcohol/cellulose nanocrystal/graphene composite aerogels (PCGAs). J. Renew. Mater..

[43] Shen D.Z., Liu J., Gan L.H., Huang N.Z., Long M.N. (2018). Green synthesis of Fe_3_O_4_/cellulose/polyvinyl alcohol hybride aerogel and its application for dye removal. J. Polym. Environ..

[44] Das G., Tesfaye R.M., Won Y., Yoon H.H. (2017). NiO-Fe_2_O_3_ based graphene aerogel as urea electrooxidation catalyst. Electrochim. Acta.

[45] Zhang X.F., Liu P., Duan Y.X., Jiang M., Zhang J.M. (2017). Graphene/cellulose nanocrystals hybrid aerogel with tunable mechanical strength and hydrophilicity fabricated by ambient pressure drying technique. RSC Adv..

[46] Chong K.Y., Chia C.H., Zakaria S., Sajab M.S., Chook S.W., Khiew P.S. (2015). CaCO_3_-decorated cellulose aerogel for removal of Congo Red from aqueous solution. Cellulose.

[47] Tang C.X., Brodie P., Li Y.Z., Grishkewich N.J., Brunsting M., Tam K.C. (2020). Shape recoverable and mechanically robust cellulose aerogel beads for efficient removal of copper ions. Chem. Eng. J..

[48] Song M.Y., Jiang J.G., Qin H.F., Ren X.Y., Jiang F. (2020). Flexible and super thermal insulating cellulose nanofibril/emulsion composite aerogel with quasi-closed pores. ACS Appl. Mater. Interfaces.

[49] Li Y.W., Jia P.P., Xu J., Wu Y., Jiang H., Li Z. (2020). The aminosilane functionalization of cellulose nanofibrils and the mechanical and CO_2_ adsorption characteristics of their aerogel. Ind. Eng. Chem. Res..

[50] Lai H.H., Zhuo H., Hu Y.J., Shi G., Chen Z.H., Zhong L.X., Zhang M.Y. (2021). Anisotropic carbon aerogel from cellulose nanofibers featuring highly effective compression stress transfer and pressure sensing. ACS Sustain. Chem. Eng..

[51] Jiang W.Z., Yao C.F., Chen W., Li D., Zhong L.X., Liu C.F. (2020). A super-resilient and highly sensitive graphene oxide/cellulose-derived carbon aerogel. J. Mater. Chem. A.

[52] Feng C.T., Ren P.G., Li Z., Tan W.Z., Zhang H., Jin Y.L., Ren F. (2020). Graphene/waste-newspaper cellulose composite aerogels with selective adsorption of organic dyes: Preparation, characterization, and adsorption mechanism. New J. Chem..

[53] Ma M.T., Chen Y.L., Zhao X., Tan F.Z., Wang Y.H., Cao Y.F., Cai W.J. (2020). Effective removal of cation dyes from aqueous solution using robust cellulose sponge. J. Saudi Chem. Soc..

[54] Dai H.J., Huang Y., Huang H.H. (2018). Eco-friendly polyvinyl alcohol/carboxymethyl cellulose hydrogels reinforced with graphene oxide and bentonite for enhanced adsorption of methylene blue. Carbohydr. Polym..

[55] Xu C.X., Jiang S., Han F.Y., Xu F., Liu L.F. (2019). Preparation of cellulose nanofibrils aerogel and its adsorption of methylene blue. J. Text. Res..

[56] Wang J.N., Yi Y., Bian Y.J., Ma Y.Y., Liu Z.M. (2019). Preparation and adsorption properties of cellulose/diatomite composite aerogel balls. J. Cellul. Sci. Technol..

[57] Xu J., Du P.F., Bi W.D., Yao G.H., Li S.S., Liu H. (2020). Graphene oxide aerogels co-functionalized with polydopamine and polyethylenimine for the adsorption of anionic dyes and organic solvents. Chem. Eng. Res. Des..

[58] Hu C.R., Li X.L., Fang Y., Yang K., Liu Y.M. (2017). Adorption properties of amaranth dye by quaternary ammonium cellulose. Environ. Pollut. Control..

[59] Jiang H.L., Chen P.H., Zhang W.B., Luo S.L., Luo X.B., Au C.T., Li M.L. (2014). Deposition of nano Fe_3_O_4_@mZrO_2_ onto exfoliated graphite oxide sheets and its application for removal of amaranth. Appl. Surf. Sci..

[60] Edet U.A., Ifelebuegu A.O. (2020). Kinetics, isotherms, and thermodynamic modeling of the adsorption of phosphates from model wastewater using recycled brick waste. Processes.

[61] Vigdorowitsch M., Pchelintsev A., Tsygankova L., Tanygina E. (2021). Freundlich isotherm: An adsorption model complete framework. Appl. Sci..

[62] Sun L.H., Wang M.R., Li W., Luo S., Wu Y., Ma C.H., Liu S.X. (2020). Adsorption separation of Cr (VI) from a water phase using multiwalled carbon nanotube-immobilized ionic liquids. ACS Omega.

[63] Sun L.H., Wang M.R., Li W., Luo S., Wu Y., Ma C.H., Liu S.X. (2020). Carbon material-immobilized ionic liquids were applied on absorption of Hg^2+^ from water phase. Environ. Sci. Pollut. Res..

